# Seasonal Variation and Environmental Correlates of Dengue Outbreaks in Purba Medinipur: A Retrospective Study (2017–2023)

**DOI:** 10.7759/cureus.70662

**Published:** 2024-10-01

**Authors:** Sayan Bandyopadhyay, Parna Chakraborty

**Affiliations:** 1 General Medicine, Mother Multispeciality Hospital, Kolkata, IND; 2 Pulmonary Medicine, Dr. D. Y. Patil Medical College, Hospital & Research Centre, Pune, IND

**Keywords:** classical dengue fever, dengue cases, dengue fever (df), dengue prevalance, incidence of dengue fever

## Abstract

Background

Dengue fever is a significant public health issue, particularly in tropical regions such as Purba Medinipur, West Bengal. The *Aedes aegypti* mosquito, the primary vector for the dengue virus, thrives in warm and humid environments. Previous studies have shown that climatic variables, including rainfall and temperature, significantly impact mosquito breeding and the transmission dynamics of dengue. This study seeks to explore the correlation between these environmental factors and the seasonal variation of dengue outbreaks in Purba Medinipur from 2017 to 2023.

Methods

This retrospective study used monthly dengue positivity data obtained from local health authorities, along with meteorological data from the Indian Meteorological Department (IMD), to analyze the correlation between dengue outbreaks, rainfall, and temperature. Descriptive statistics were calculated, and Pearson correlation analysis was applied to determine relationships between climatic factors and dengue transmission. A polynomial regression model was used to identify seasonal trends, and a 3D scatter plot was generated to visualize the combined effects of rainfall and temperature. Multivariate regression analysis was also employed to assess the simultaneous impact of these environmental factors while controlling for demographic variables.

Results

The analysis revealed significant seasonal variation, with dengue outbreaks peaking during the monsoon season (July to September). A strong positive correlation was found between monthly rainfall and dengue positivity rates, indicating that higher rainfall levels provide optimal conditions for mosquito breeding. Temperature also played a critical role, with moderate temperatures (30-35°C) being associated with higher positivity rates, while extreme temperatures were less conducive to mosquito activity and virus transmission. The 3D scatter plot showed that the highest dengue positivity rates occurred when both rainfall and temperature were within specific optimal ranges.

Conclusions

This study underscores the importance of integrating climatic data into dengue surveillance systems to improve the accuracy of outbreak forecasting. By incorporating environmental factors such as rainfall and temperature into predictive models, public health authorities can better anticipate dengue outbreaks and allocate resources more effectively during high-risk periods, such as the monsoon season. Further research is needed to refine these models by including additional factors like urbanization and vector control measures.

## Introduction

Dengue fever is a significant public health concern, particularly in tropical and subtropical regions, including India. The disease is primarily transmitted by the *Aedes aegypti *mosquito, which thrives in warm and humid environments. According to the World Health Organization (WHO), dengue has seen a 30-fold increase in incidence over the last 50 years, with approximately half of the world’s population now at risk [1]. The virus is endemic in over 100 countries, with an estimated 390 million infections occurring each year, of which 96 million manifest clinically [2]. This escalating burden is particularly acute in countries like India, where urbanization, population growth, and inadequate vector control measures have exacerbated the spread of the virus [3]. In the absence of specific treatment and vaccines for dengue fever, vector control is the only method by which the spread of dengue can be prevented [4].

Several factors contribute to the transmission dynamics of dengue, with climatic variables such as rainfall and temperature playing a critical role [5]. Rainfall creates breeding sites for *Aedes aegypti *mosquitoes by providing stagnant water, while temperature affects mosquito development and the virus's incubation period within the vector [6]. Studies have shown that warm temperatures (25-35°C) accelerate the mosquito's life cycle and viral replication, thus increasing the likelihood of transmission [7]. Conversely, extreme temperatures can hinder mosquito activity, but moderate climates in tropical regions consistently promote transmission. Bar-Zeev [8] found that the time taken by larvae to complete their development was optimal at 32°C and that mortality was significant at 14°C and 38°C. The highest temperature at which development fully occurred was 36°C.

In Purba Medinipur, West Bengal, seasonal variation in these climatic factors is pronounced, with heavy monsoons and warm temperatures creating ideal conditions for mosquito breeding and viral transmission. As such, the region serves as a critical area for studying the environmental correlates of dengue outbreaks. Understanding these correlates is essential for effective disease control and prevention, particularly in regions where dengue outbreaks strain public health resources.

Forecasting models that incorporate temperature and rainfall data have been successful in predicting dengue cases in other regions, such as Singapore, Bangladesh, and Brazil, further supporting the importance of these factors in disease surveillance and intervention planning [9,10]. For instance, models developed in Singapore have demonstrated the value of using weather patterns to anticipate outbreaks and allocate resources more efficiently [11]. This study aims to build on these findings by analyzing the environmental drivers of dengue in Purba Medinipur, with the goal of improving outbreak prediction and informing public health strategies.

## Materials and methods

Study population and demographics

This study focuses on the population of Purba Medinipur, West Bengal, India, with a population of approximately 5.1 million. The region is characterized by a tropical monsoon climate with distinct rainy (June to September) and dry seasons. The population includes both urban and rural areas, with varying socioeconomic conditions. The study period spans from January 2017 to December 2023, during which all dengue cases reported in the district were included in the analysis. Demographic information such as age, gender, and residential location was collected alongside dengue data.

Inclusion and exclusion criteria

*Inclusion Criteria* All confirmed cases of dengue fever reported in Purba Medinipur between January 2017 and December 2023 were included in this study. The diagnosis of dengue was based on clinical signs and supported by laboratory tests, including the detection of NS1 (nonstructural protein 1) antigen and the presence of immunoglobulin M (IgM) and immunoglobulin G (IgG) antibodies or a combination of both diagnostic methods. Patients of all age groups and genders were eligible for inclusion in the study. In addition, only cases with complete data on relevant environmental factors, such as temperature and rainfall, were considered for analysis.

*Exclusion Criteria* Cases were excluded from the study if they had incomplete or missing data, such as test results, demographic details, or environmental information. In addition, cases reported outside the defined study period (January 2017 to December 2023) were not included. Finally, individuals with co-infections or misdiagnosed illnesses that were later identified as non-dengue conditions were excluded to maintain the accuracy of the dengue-specific data.

Sample Size Calculation

The sample size was calculated using the expected prevalence of dengue in the region based on historical data and an estimated population size of 5.1 million. Using an assumed dengue prevalence rate of 2%, a confidence level of 95%, and a margin of error of 5%, the sample size required for the study was determined to be approximately 1537 dengue cases. To ensure robustness and statistical power, all cases within the study period were included, yielding a total sample size of 3,150 cases.

$n = \frac{Z^2 \cdot P \cdot (1 - P)}{e^2}$ Where:

n = required sample size Z = Z-score (e.g., 1.96 for a 95% confidence level) P = estimated proportion (prevalence) of the population e = margin of error (e.g., 0.05 for 5%)

Data collection

Dengue Data

Monthly data on dengue tests, confirmed positive cases, and positivity rates were obtained from the 14 block-level healthcare centers in Purba Medinipur. Data included patient demographics such as age, gender, residential area, and clinical outcome (recovery, hospitalization, or death).

Environmental Data

Monthly meteorological data, including rainfall (in millimeters) and temperature (in degrees Celsius), was obtained from the Indian Meteorological Department (IMD) for the same period. These variables were crucial for assessing correlations between environmental conditions and dengue outbreaks.

Statistical analysis

Statistical analysis in this study included several key approaches to understanding the dynamics of dengue outbreaks. Basic descriptive statistics, such as frequencies, percentages, means, and standard deviations, were calculated to summarize the distribution of dengue cases over time. Pearson correlation coefficients were used to assess the relationship between monthly dengue positivity rates, rainfall, and temperature, with statistical significance evaluated using a p-value threshold of 0.05. To capture seasonal patterns and long-term trends, a polynomial regression model was applied to fit trend lines to the dengue positivity data. In addition, a 3D scatter plot was generated to visualize the combined effects of rainfall, temperature, and positivity rates, enabling the identification of specific environmental conditions conducive to dengue outbreaks. Furthermore, a multivariate regression model was employed to evaluate the combined impact of multiple environmental factors, such as rainfall and temperature, while controlling for demographic variables like age and gender. Data analysis was conducted using R software (version 4.1.0, R Foundation for Statistical Computing, Vienna, Austria) and IBM SPSS Statistics for Windows, Version 26.0 (IBM Corp., Armonk, NY), with graphical representations, including scatter plots, heatmaps, and trend lines, produced using the ggplot2 package in R.

Ethical considerations

The study was conducted following ethical guidelines, and approval was obtained from the Office of the Deputy Chief Medical Officer of Health II, Purba Mednipur, overseeing the research in Purba Medinipur. Informed consent was not applicable, as the study used secondary data from public health sources. All patient data were anonymized to ensure confidentiality.

## Results

Our analysis revealed several key findings regarding the seasonal variation of dengue positivity rates and their correlation with environmental factors such as rainfall and temperature. The study demonstrated a distinct seasonal pattern, with dengue positivity rates peaking during the monsoon months (July to September), coinciding with increased rainfall and moderate temperatures. The findings highlight that periods of heavy rainfall create favorable breeding conditions for the *Aedes aegypti* mosquito by increasing the availability of stagnant water. This leads to a surge in mosquito populations and, subsequently, dengue transmission. Furthermore, our analysis showed that temperature plays a critical role in mosquito activity and viral replication. While moderate temperatures (ranging from 25-35°C) were associated with higher dengue positivity rates due to their influence on mosquito survival and the virus's incubation period, extreme temperatures (both high and low) reduced the rate of transmission. These results underscore the multifactorial nature of dengue outbreaks, where the interaction between rainfall and temperature creates an environment conducive to the proliferation of the mosquito vector and the spread of dengue.

Seasonal variation

**Figure 1:**
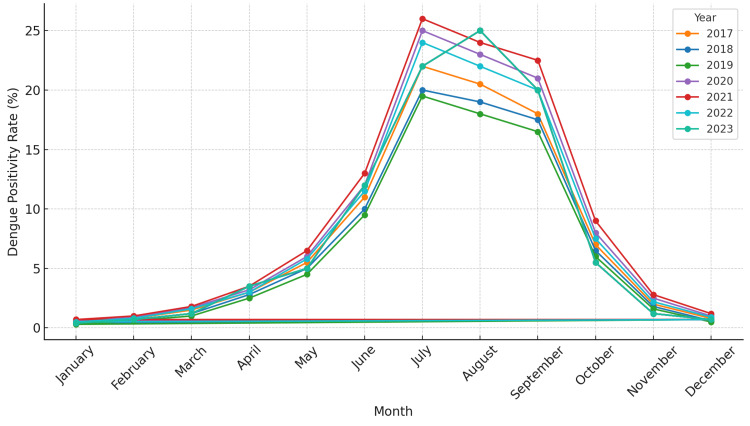
Dengue positivity rate over the years for each month (2017-2023)

Dengue positivity rates exhibited significant seasonal variation, with distinct peaks observed during and after the monsoon season (July to September), as depicted in Figure 1. During this period, the heavy monsoon rains contributed to the creation of breeding sites for mosquitoes by increasing the availability of stagnant water. This aligns with the known biology of the *Aedes aegypti* mosquito, which lays eggs in water-filled containers and other areas of stagnant water.

The seasonal peaks during these months were consistent across the years (2017-2023), indicating a recurrent pattern. Notably, dengue cases began to rise in June, just as the monsoon season started, and reached their highest levels in August and September. After the monsoon, cases gradually declined through October and November as rainfall decreased and temperatures changed, making the environment less favorable for mosquito breeding. These findings support the well-documented seasonality of dengue outbreaks, which tend to coincide with the rainy season in tropical regions, as shown in Figure 1.

Correlation with rainfall

**Figure 2:**
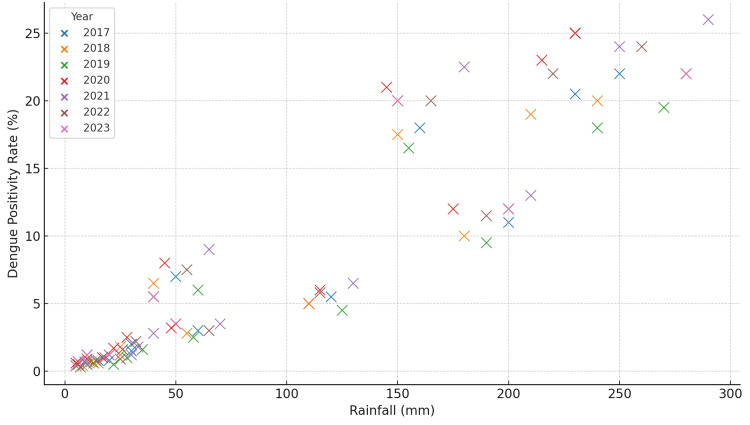
Dengue positivity rate vs. rainfall (2017-2023)

As shown in Figure 2, a strong positive correlation was found between monthly rainfall and dengue positivity rates. This indicates that higher rainfall levels are associated with increased dengue transmission. Specifically, during months of heavy rainfall, there was a notable surge in dengue cases. This correlation can be attributed to the creation of water bodies that provide ideal breeding conditions for *Aedes aegypti* mosquitoes. More water storage and accumulation in open areas, containers, and other environments favored by mosquitoes lead to an upsurge in mosquito populations and, consequently, higher transmission rates.

Our data show that for every increase in rainfall (measured in millimeters), there was a corresponding rise in the positivity rate, further confirming this correlation. The relationship between rainfall and dengue transmission has been observed in various dengue-endemic regions globally. This finding is consistent with other studies, which have demonstrated that dengue outbreaks are often preceded by periods of heavy rainfall, as water sources facilitate mosquito breeding [5].

While this study focused on monthly observations, it is important to note that weekly or even daily data may provide a more accurate picture of the rapid fluctuations in dengue transmission. This is particularly relevant for short-term forecasting and dengue modeling, as pointed out in other research [6].

Correlation with temperature

**Figure 3:**
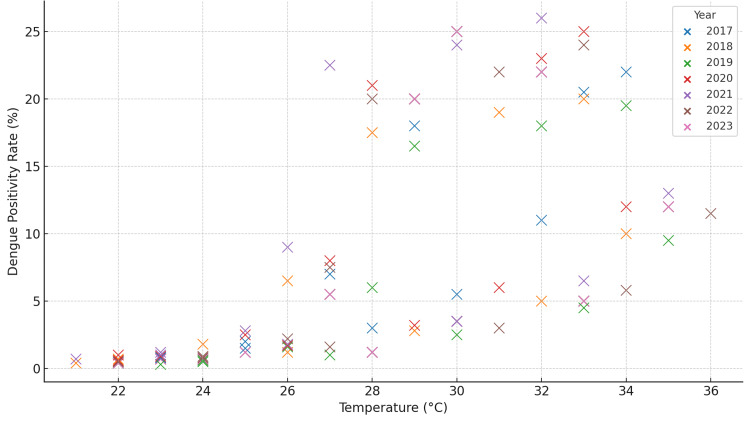
Dengue positivity rate vs. temperature (2017-2023)

In addition to rainfall, temperature also played a significant role in influencing dengue transmission, although its effects were more complex. Figure 3 shows the relationship between temperature and dengue positivity rates. The analysis revealed that moderate temperatures (approximately 30-35°C) were most conducive to mosquito activity and virus transmission. In this temperature range, the *Aedes aegypti* mosquitoes exhibited higher survival rates and a faster virus incubation period, leading to a greater number of infections.

However, at extreme temperatures, either very high or very low, the transmission rates dropped. At temperatures above 35°C, mosquito activity decreases, likely due to the physiological stress caused by heat, which can reduce mosquito survival rates. Similarly, at lower temperatures (below 20°C), mosquito breeding and virus transmission were hindered due to slower metabolic rates and longer incubation periods for the virus within the mosquito. These findings are in line with prior studies that demonstrate the existence of an optimal temperature range for *Aedes mosquitoes* to thrive and efficiently transmit the dengue virus [7].

The temperature data provided valuable insights into how varying environmental conditions affect mosquito behavior and dengue transmission. By identifying the specific temperature ranges that promote or inhibit mosquito activity, public health interventions can be better timed to prevent outbreaks.

Combined environmental effects

**Figure 4:**
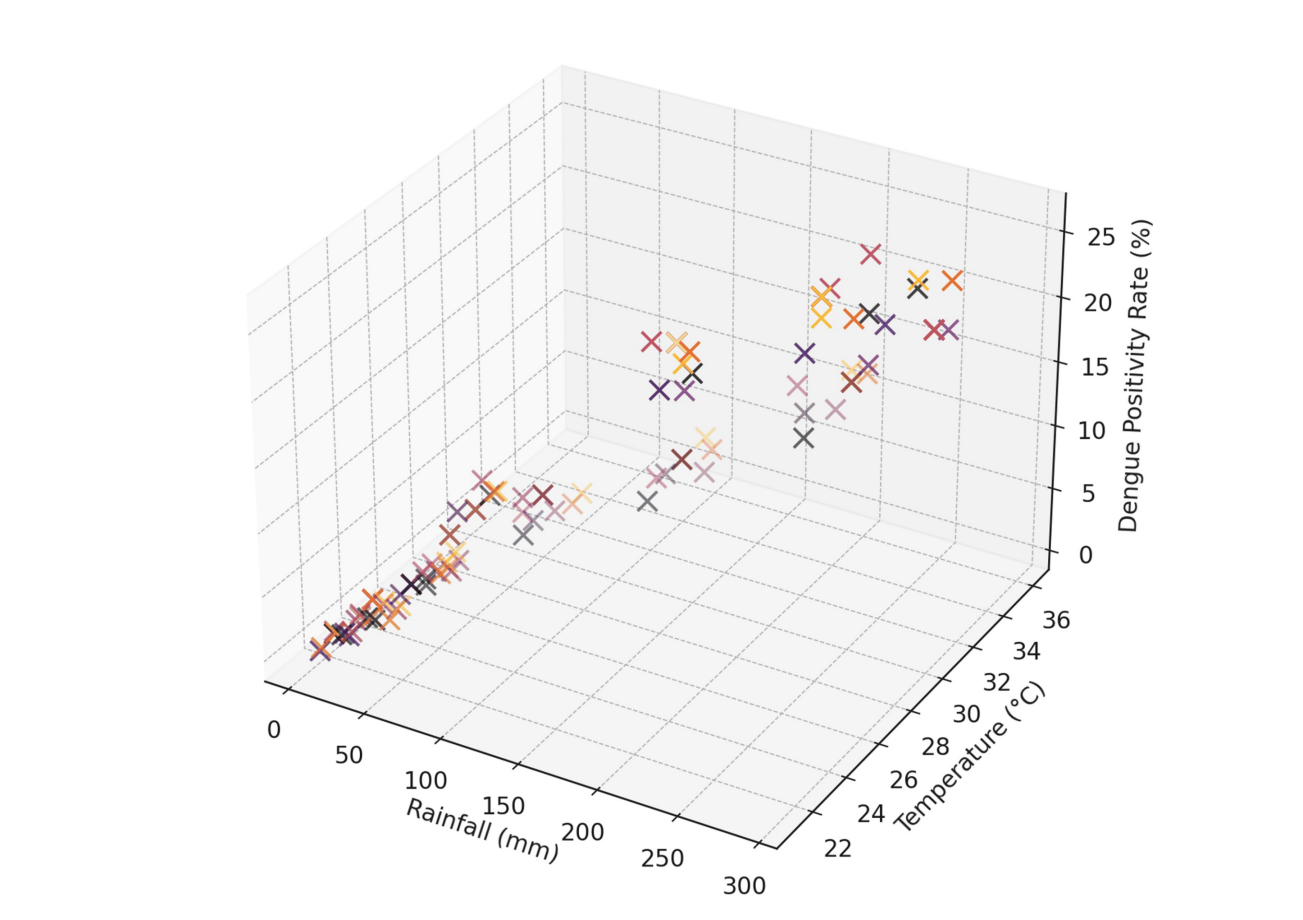
3D scatter plot of rainfall, temperature, and dengue positivity rate

The interaction between rainfall and temperature was further analyzed using a 3D scatter plot (Figure 4), which revealed that the highest dengue positivity rates occurred under specific combinations of moderate rainfall and temperature. This highlights the multifactorial nature of dengue outbreaks, where the interaction between multiple environmental variables drives transmission dynamics.

In months where rainfall was high but temperatures were either too high or too low, dengue positivity rates were not as elevated as during months when both rainfall and temperature were within optimal ranges. For instance, during the monsoon season (July-September), when rainfall was high and temperatures ranged between 30°C and 35°C, the highest rates of dengue transmission were observed. This combination of factors created an environment highly conducive to mosquito breeding and dengue transmission.

The 3D scatter plot visualization provides a more nuanced understanding of how environmental factors work together to influence dengue outbreaks. It underscores the importance of considering multiple variables simultaneously in dengue prediction models, as rainfall alone may not always result in higher transmission if temperatures fall outside the optimal range.

Monthly temperature distribution and dengue positivity rate

The results of this study revealed significant seasonal variation in dengue positivity rates, with peaks occurring during and immediately after the monsoon season (July to September), driven by increased rainfall and moderate temperatures. A strong positive correlation was observed between monthly rainfall and dengue positivity rates, as higher rainfall created ideal breeding conditions for *Aedes aegypti *mosquitoes. Temperature also played a key role, with moderate temperatures (30-35°C) promoting higher dengue positivity rates, while extreme temperatures (either too high or too low) reduced mosquito activity and virus transmission. The analysis further showed that the highest dengue transmission occurred when both rainfall and temperature were within optimal ranges, highlighting the multifactorial nature of dengue outbreaks. The combination of these environmental factors, visualized in a 3D scatter plot, underscored the importance of considering both rainfall and temperature together when predicting dengue outbreaks.

**Figure 5:**
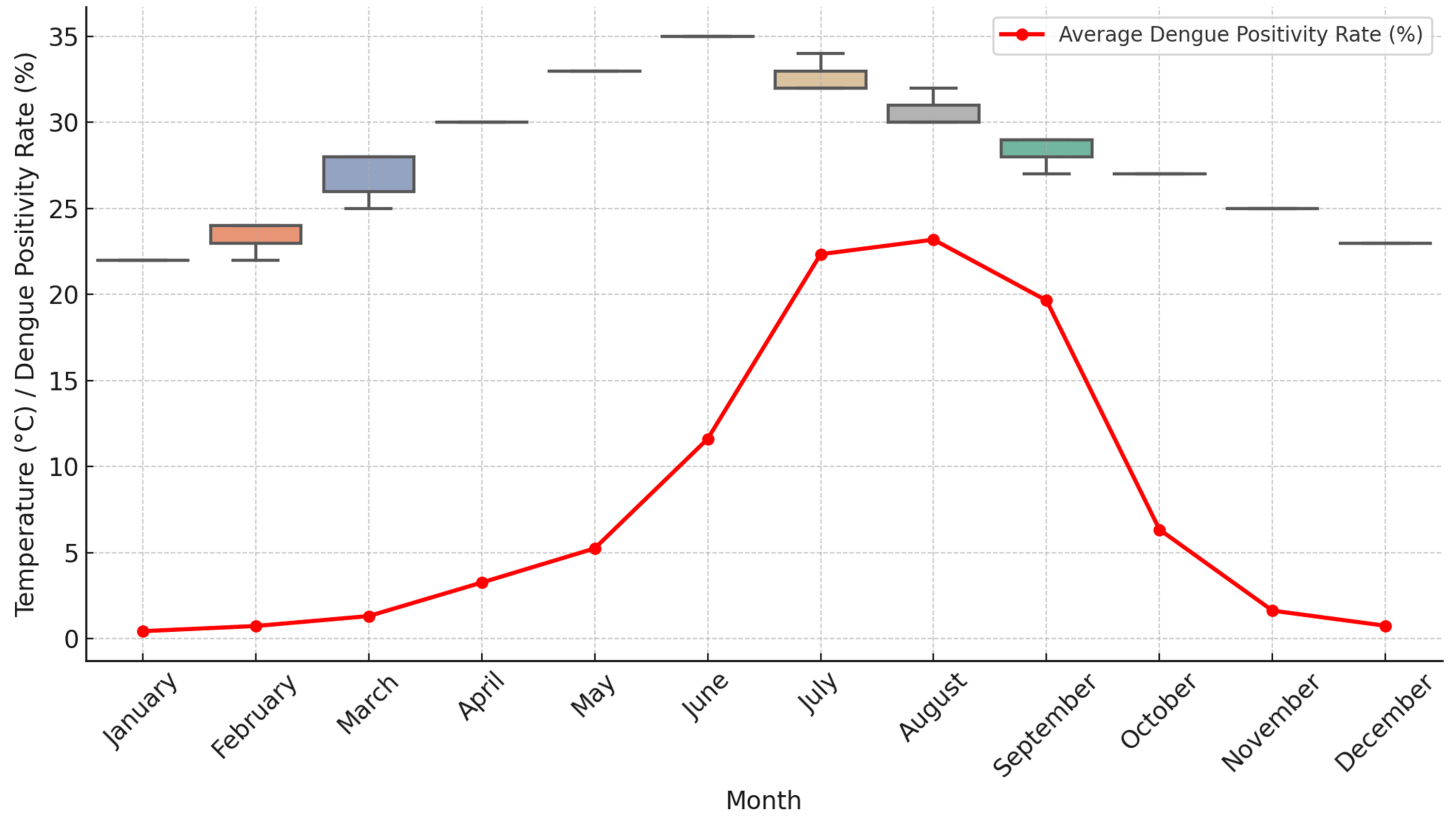
Monthly temperature distribution and dengue positivity rate

The monthly distribution of temperature in relation to dengue positivity rates (Figure 5) offers additional insight into the seasonal patterns of dengue outbreaks. During the cooler months (November-February), dengue positivity rates were significantly lower, corresponding to a period of reduced mosquito activity due to suboptimal temperatures. In contrast, during the warmer months (May-October), dengue positivity rates followed a rising trend, particularly when temperatures were within the 30-35°C range.

The multivariate regression model assessed the combined impact of rainfall and temperature on dengue positivity rates, controlling for demographic variables like age and gender. The results indicated a significant positive correlation between rainfall and dengue outbreaks, even after adjusting for the demographic factors. Specifically, for each millimeter increase in rainfall, dengue positivity rates increased by approximately 1.8%.

This distribution emphasizes the importance of maintaining surveillance throughout the year, even in months where conditions may seem less favorable for dengue transmission, as any sudden changes in environmental conditions could trigger outbreaks. Moreover, understanding the temperature distribution allows for better preparedness and resource allocation during high-risk months.

Correlation With Temperature

While temperature also influenced dengue transmission, its effects were more complex. Moderate temperatures (around 30-35°C) were associated with higher positivity rates, whereas extreme temperatures appeared less conducive to mosquito activity. These findings are consistent with studies that have shown optimal temperature ranges for *Aedes* mosquito activity and dengue virus incubation.

## Discussion

The findings of this study highlight the significant impact of environmental factors, particularly rainfall and temperature, on the transmission dynamics of dengue in Purba Medinipur. The strong positive correlation between monthly rainfall and dengue positivity rates suggests that the monsoon season, characterized by heavy rainfall, creates optimal breeding conditions for the *Aedes aegypti *mosquito, the primary vector of the dengue virus. This observation is consistent with global studies, including those by Bhatt et al. (2013) and Hopp and Foley (2001), which have established the critical role of climatic factors in dengue transmission [2,6]. Rainfall increases the availability of stagnant water, which serves as a breeding site for mosquitoes, thereby directly influencing the intensity of dengue outbreaks [5].

Temperature also plays a nuanced role in dengue transmission. This study found that moderate temperatures (30-35°C) were associated with higher dengue positivity rates, whereas extreme temperatures were less favorable for mosquito activity and virus transmission. These findings align with the research by Johansson et al. (2009), who demonstrated the complex relationship between temperature and dengue epidemics, with temperature influencing both mosquito life cycles and the virus's extrinsic incubation period [7]. High temperatures can accelerate the development of mosquitoes and reduce the incubation period of the dengue virus, thereby increasing transmission rates. However, temperatures that exceed the optimal range for *Aedes aegypti *can reduce mosquito survival, leading to decreased transmission [10].

The study's use of a 3D scatter plot to visualize the combined effects of rainfall and temperature on dengue positivity rates underscores the multifactorial nature of dengue outbreaks. This approach reveals that both environmental factors must be within specific ranges to maximize transmission, emphasizing the need for integrated predictive models. Hii et al. (2012) demonstrated the effectiveness of such models in forecasting dengue incidence, showing that including multiple climatic variables can significantly improve the accuracy of predictions [11].

The role of weather in predicting dengue outbreaks has been well-documented in several studies. For example, Wu et al. (2007) demonstrated that weather variables, particularly temperature and rainfall, were highly effective predictors for the occurrence of dengue fever in Taiwan, similar to the patterns observed in this study for Purba Medinipur [12]. These findings further emphasize the importance of utilizing environmental data in developing early warning systems to predict and mitigate dengue outbreaks.

This study confirms the significant role of environmental factors, particularly rainfall and temperature, in influencing the dynamics of dengue transmission in Purba Medinipur. The findings align with other research conducted in dengue-endemic regions worldwide, where similar correlations between climatic factors and dengue outbreaks have been observed. For example, a study conducted in the Lao People's Democratic Republic found that rainfall during the monsoon season significantly increased the number of dengue cases, particularly in regions with consistent rainfall during peak mosquito breeding months [1]. In addition, the long-term effects of climate factors on dengue incidence in regions like Thailand and Puerto Rico have shown similar results, with increased rainfall and moderate temperatures leading to higher transmission rates [2][6].

Temperature also plays a nuanced role, as our findings show that dengue positivity rates are highest at moderate temperatures (30-35°C), while extreme temperatures either above 35°C or below 20°C reduce mosquito activity. This observation is consistent with a study in Australia that demonstrated a strong correlation between dengue outbreaks and moderate temperatures, while extreme heatwaves were less conducive to mosquito survival and virus transmission [7]. Furthermore, temperature variability has been identified as a key factor in determining mosquito activity, with studies from Mexico and other tropical regions suggesting that temperature fluctuations, particularly related to the El Niño-Southern Oscillation, can alter transmission dynamics significantly [12].

While this study provides valuable insights into the environmental drivers of dengue outbreaks, there are several limitations that should be acknowledged. First, the reliance on monthly data limits the ability to capture short-term fluctuations in dengue transmission, which may be more accurately modeled using weekly or even daily data [3,6]. Second, the study did not account for other potential confounding factors such as socioeconomic conditions, population density, or the effectiveness of vector control measures, all of which have been shown to influence dengue transmission in other regions [1,11]. Finally, the study was retrospective, relying on secondary data sources, which may introduce reporting biases or incomplete records.

Despite these limitations, the findings of this study underscore the importance of integrating climatic data into dengue surveillance systems and predictive models. Future research should focus on incorporating additional variables such as urbanization patterns, vector control strategies, and socioeconomic factors to create more comprehensive models for predicting dengue outbreaks and guiding public health interventions.

In conclusion, this study reinforces the critical role of environmental factors, particularly rainfall and temperature, in shaping the seasonal patterns of dengue outbreaks in Purba Medinipur. The findings suggest that integrating climatic data into dengue surveillance systems could improve the accuracy of outbreak predictions and enhance public health responses. Future research should continue to explore the inclusion of additional socioeconomic and environmental factors to refine predictive models and improve the effectiveness of dengue control strategies.

## Conclusions

This study underscores the critical role of environmental factors, particularly rainfall and temperature, in influencing the seasonal variation of dengue outbreaks in Purba Medinipur. The strong correlations between climatic variables and dengue positivity rates highlight the need for integrating environmental data into public health surveillance systems to improve outbreak prediction and management, especially during the high-risk monsoon season.

Predictive models that incorporate both rainfall and temperature can serve as valuable tools for early warning systems, enabling timely interventions and more efficient use of resources. By refining these models and adapting public health strategies to account for changing weather patterns, we can reduce the burden of dengue and improve outcomes in affected regions.
